# The comprehensive English National Lynch Syndrome Registry: development and description of a new genomics data resource

**DOI:** 10.1016/j.eclinm.2024.102465

**Published:** 2024-02-07

**Authors:** Catherine Huntley, Lucy Loong, Corinne Mallinson, Rachel Bethell, Tameera Rahman, Neelam Alhaddad, Oliver Tulloch, Xue Zhou, Jason Lee, Paul Eves, GMSA Lynch Consortium, Jacqueline Cook, Ruth Armstrong, Munaza Ahmed, Terri McVeigh, Bianca DeSouza, Anjana Kulkarni, Heirdre Bezuidenhout, Richard Martin, Debbie Holliday, Rachel Hart, Fiona Lalloo, Alan Donaldson, Ruth Cleaver, Catherine Willis, Victoria Kiesel, Marie-Anne O'Reilly, Dorothy Halliday, Joyce Solomons, Kai Ren Ong, Fiona McRonald, Bethany Torr, John Burn, Adam Shaw, Eva J.A. Morris, Kevin Monahan, Steven Hardy, Clare Turnbull

**Affiliations:** a Division of Genetics and Epidemiology, The Institute of Cancer Research, Sutton, UK; b National Disease Registration Service, NHS England, London, UK; c Health Data Insight CIC, Cambridge, UK; d Translational and Clinical Research Institute, Newcastle University, Newcastle, UK; e Guy's and St Thomas' NHS Foundation Trust, London, UK; f Big Data Institute, Nuffield Department of Population Health, University of Oxford, Oxford, UK; g The Lynch Syndrome and Family Cancer Clinic, St Mark's Hospital and Academic Institute, Harrow, London, UK; h Imperial College London, London, UK

**Keywords:** Lynch syndrome, Registry, Genomics, Data

## Abstract

**Background:**

Lynch Syndrome (LS) is a cancer predisposition syndrome caused by constitutional pathogenic variants in the mismatch repair (MMR) genes. To date, fragmentation of clinical and genomic data has restricted understanding of national LS ascertainment and outcomes, and precluded evaluation of NICE guidance on testing and management. To address this, via collaboration between researchers, the National Disease Registration Service (NDRS), NHS Genomic Medicine Service Alliances (GMSAs), and NHS Regional Clinical Genetics Services, a comprehensive registry of LS carriers in England has been established.

**Methods:**

For comprehensive ascertainment of retrospectively identified MMR pathogenic variant (PV) carriers (diagnosed prior to January 1, 2023), information was retrieved from all clinical genetics services across England, then restructured, amalgamated, and validated via a team of trained experts in NDRS. An online submission portal was established for prospective ascertainment from January 1, 2023. The resulting data, stored in a secure database in NDRS, were used to investigate the demographic and genetic characteristics of the cohort, censored at July 25, 2023. Cancer outcomes were investigated via linkage to the National Cancer Registration Dataset (NCRD).

**Findings:**

A total of 11,722 retrospective and 570 prospective data submissions were received, resulting in a comprehensive English National Lynch Syndrome Registry (ENLSR) comprising 9030 unique individuals. The most frequently identified pathogenic MMR genes were *MSH2* and *MLH1* at 37.2% (n = 3362) and 29.1% (n = 2624), respectively. 35.9% (n = 3239) of the ENLSR cohort received their LS diagnosis before their first cancer diagnosis (presumptive predictive germline test). Of these, 6.3% (n = 204) developed colorectal cancer, at a median age of initial diagnosis of 51 (IQR 40–62), compared to 73 years (IQR 64–80) in the general population (p < 0.0001).

**Interpretation:**

The ENLSR represents the first comprehensive national registry of PV carriers in England and one of the largest cohorts of MMR PV carriers worldwide. The establishment of a secure, centralised infrastructure and mechanism for routine registration of newly identified carriers ensures sustainability of the data resource.

**Funding:**

This work was funded by the 10.13039/100010269Wellcome Trust, 10.13039/501100000289Cancer Research UK and 10.13039/100013791Bowel Cancer UK. The funder of this study had no role in study design, data collection, data analysis, data interpretation, or writing of the report.

**Research in context**

**Evidence before this study**

We searched the PubMed database for articles containing the terms “Lynch Syndrome” or “Hereditary Cancer” and “Registry” from 1985 to 2023. There are smaller single-country hereditary cancer registries and a large prospective research database comprising patients voluntarily recruited from multiple countries with heterogeneous and incomplete case ascertainment, and variable surveillance and management. There are also many series of Lynch Syndrome patients reported which are largely voluntarily recruited, small in size and retrospectively ascertained, and therefore vulnerable to biases.

**Added value of this study**

We present here a description of the first national retrospectively and prospectively comprehensive genomic rare disease registry in the UK, and one of the largest known cohorts of MMR pathogenic variant carriers, providing unique insight into the size, demographics and genetic characteristics of the Lynch Syndrome population in England. The English National Lynch Syndrome Registry represents a growing, sustainable and valuable resource to support delivery of high-risk cancer screening, service evaluation, identification of clinical trial participants and cancer outcomes research.

**Implications of all the available evidence**

Patient registries are a useful tool for systematic service provision and audit which can help identify and address inequities. Patient registries may also be used as cohorts to support research into epidemiology, cancer prevention, management and outcomes, to the benefit of the Lynch Syndrome population. The structure, governance and novel methodology to assemble the English National Lynch Syndrome Registry, serve as an exemplar for the establishment of other patient registries for cancer susceptibility syndromes or rare diseases.

## Introduction

Recognising the challenges in improving outcomes in advanced cancers, renewed emphasis has been placed on reducing cancer burden via early detection, screening, and prevention (SPED), as articulated in the NHS England Long Term Plan.^1^ Concurrently, there has also been a stronger emphasis on the role of genomics within routine NHS clinical care, delivered through the Genomic Medicine Service (GMS). Whilst historically limited to population-level screening, the remit of the UK National Screening Committee (UKNSC) has recently been extended to include targeted screening programs for population subgroups at elevated risk. Positioned at the intersection of these three broad initiatives are the genetic syndromes of high penetrance cancer susceptibility, of which Lynch Syndrome (LS) is a well-characterised archetype.

Clustering within families of cases of gastrointestinal and gynaecological cancers was reported more than 100 years ago, an association subsequently termed Lynch Syndrome (LS).^2^ Analyses of genome-wide markers in modest numbers of multi-case families led to successful linkage mapping of the high-penetrance genes *MLH1* and *MSH2* genes, with two further LS genes *MSH6* and *PMS2* identified via association studies of additional genes also involved in mismatch repair (MMR). Longstanding European national LS registries, several of which were established before identification of the MMR genes, were established with the aim of organising nationwide screening and management of families with hereditary colorectal cancer phenotypes including Lynch Syndrome, and were critical, alongside voluntary research cohorts, in establishing cancer mitigation strategies.^3^, ^4^, ^5^ Evidenced strategies for individuals carrying LS pathogenic variants, include colonoscopic surveillance, preventative polypectomy, aspirin chemoprophylaxis and gynaecological surgical prophylaxis.^6^, ^7^, ^8^ International collaborative efforts to pool prospectively followed patients with LS have yielded informative cumulative gene and gender specific cancer risk estimates.^9^

Due to genomic technologies previously being costly and of low throughput, testing for LS genes was historically restricted to affected probands from multi-case families, with eligibility delineated on the basis of complex criteria of personal or family cancer history. However, over the last decade, advances in next-generation sequencing technologies have enabled dramatic expansion of genomic analyses with concomitant reduction in cost. Following guidance issued by the National Institute for Health and Care Excellence (NICE), eligibility for LS testing was extended in 2017 to include all incident cases of colorectal cancer, and in 2020 all incident cases of endometrial cancer.^10^^,^^11^ Based on population mutational frequency, disease penetrance, and efficacy of interventions, along with the hereditary breast ovarian cancer genes (e.g., *BRCA1* and *BRCA2*) and familial hypercholesterolaemia genes (e.g., *LDLR*), LS testing is widely agreed to be one of the genetic tests of highest clinical and public health utility.

However, whilst genomic sequencing technologies have advanced rapidly, national organisation of health records and clinical genomic data have remained fragmented, typically involving locally held lists and laboratory databases. Accordingly, it has, to date, been challenging to evaluate implementation of the NICE guidance, assess national ascertainment of LS carriers, investigate consistency of clinical care and outcomes, or locate patients for clinical trials. Hence, a collaborative national endeavour involving researchers, the National Disease Registration Service (NDRS), NHS Genomic Medicine Service Alliances (GMSAs), and all NHS Regional Clinical Genetics Services, has been undertaken to amalgamate all MMR pathogenic variant (PV) carriers and to establish a prospective mechanism for routine national centralised registration of newly identified LS carriers. We present here descriptive analysis of the English National Lynch Syndrome Registry (ENLSR).

## Methods

### Establishment of the English National Lynch Syndrome Registry

A common data model was created in collaboration with NDRS and GMSA partners to capture key demographic and genetic data on all known MMR PV carriers in England (Supplementary Table 1). The common data model was used for collecting both retrospective and prospective data, and served as the storage structure of the data within NDRS.

For retrospective data collection, all English Regional Clinical Genetics Services were contacted and requested to submit data on all MMR PV carriers identified by their service, from the inception of their service until January 1, 2023. They were supplied with the common data model as an exemplar, but no limits were placed on the type of data accepted. The data were received via secure batch submissions from all English Regional Clinical Genetics Services. The data were received in a variety of formats, including free text laboratory reports. A process of automated data extraction, restructuring to the common data model, and amalgamation into a single file was piloted, but replaced by a manual process using a group of trained experts due to erroneous and missing data items identified on automated extraction. Where concordance was achieved on data extraction by two independent reviewers, and data was assessed to be of sufficient quality (no clear and obvious errors), submissions were judged to have passed genetic data validation and progressed to demographic data validation. Where submissions failed genetic data validation, a request for further detail was sent to the referring centre. Responses to requests were again passed through the genetic data validation process, and those not achieving concordant extraction or quality standards were excluded.

Demographic data was validated by automated matching against the NHS Spine, a demographic and healthcare database supporting the provision of NHS care in England.^12^ Where submissions matched a record in the NHS Spine by either a combination of i) NHS Number and date of birth or ii) a combination of first name, last name, date of birth, gender, and post code, the submission was considered to have passed demographic data validation and any missing or outdated demographic information from the original submission was updated with information extracted from the NHS Spine. Submissions that could not be automatically traced were manually searched for in the NHS Spine by two independent data managers. Where both data managers independently identified the same match for a submission, the demographic validation check was accepted. Submissions that could not be automatically or manually traced were returned to their source with a request for clarification. Responses were passed through the demographic data validation process again, and those failing this second round of data validation were excluded. Data were de-duplicated and uploaded into the ENLSR database.

To facilitate the submission of prospective data on newly identified LS patients by their responsible clinician, new functionality was developed for the secure online NDRS API portal. This allowed data to be structured at the point of entry in line with the common data model. Validations were built into the portal such that data not meeting the structural requirements of the datasets (i.e., non-MMR gene, or missing digit in NHS number) cannot be submitted, and will result in an error being returned to the submitting clinician indicating that they should re-enter the information. Demographic data are automatically traced and validated, and data is directly uploaded into the ENLSR. The online portal was activated on January 1, 2023 and submissions collected after this date are considered prospective (Supplementary Fig. 1, Supplementary Methods).

The full contents of the ENLSR were downloaded on July 25, 2023 (all years for which data were available), for use in this analysis. Unique individuals were identified on the basis of NHS Number and date of birth. Where duplicate entries were identified with the same NHS Number and date of birth but inconsistent accompanying data, inconsistencies were resolved using the variable-specific conflict resolution rules described in Supplementary Table 2.

### Analysis of the English National Lynch Syndrome Registry and linked cancer data

Descriptive analysis of the ENLSR was performed using R (v4.3.1). We included pathogenic *EPCAM* variants (n = 65) in analyses under the *MSH2* category. Age in years was calculated to July 25, 2023, excluding individuals who were deceased on or before that date. Presumptive test scope was determined on the basis of the temporal relationship between date of LS diagnosis and first cancer diagnosis: predictive (no cancer diagnoses, or LS diagnosis >60 days before first cancer diagnosis), diagnostic (LS diagnosis at time of (±60 days) or after first cancer diagnosis) or unknown (temporal relationship could not be established due to missing LS diagnosis date, or LS diagnosis date after latest date for which cancer registration data were available (December 31, 2020)). Population and geography data from the National Cancer Registration and Analysis Service (NCRAS) and ONS's Open Geography portal were used to map the postcode of residence of MMR PV carriers to Cancer Alliances and retrieve total population counts for Cancer Alliances.^13^ (A summary of data sources used can be found in Supplementary Table 3).

Data were linked via NHS number to the National Cancer Registration Dataset (NCRD) maintained by NCRAS.^14^ We extracted data on all cancers occurring in the LS registry cohort between January 1, 1995, and December 31, 2020 (all years for which finalised cancer registration data were available), and performed descriptive analyses, using the ICD-10 codes recorded in the NCRD to classify cancer types. We used ICD-10 codes to classify cancer types as follows: Upper GI (C15, C16, C17, C22, C23, C24, C25), Colorectal (C18, C19, C20), Ovarian (C48, C56, C57), Endometrial (C54), and Urinary tract (C64, C65, C66, C67, C68). To facilitate comparison we also extracted data on all cancers occurring in the general population between January 1, 1995, and December 31, 2020 and subtracted individuals who are in the ENSLR (Supplementary Methods).

Data classifying tumours diagnosed between 2006 and 2018 into one of eight routes to diagnosis (screen-detected, two week wait, emergency presentation, GP referral, inpatient elective, other outpatient, death certificate only, and unknown) was supplied by NCRAS.^15^^,^^16^ No further years of route to diagnosis data were available. Due to small numbers, GP referral, inpatient elective, and other outpatient were collapsed into a single ‘routine’ category; while death certificate only and unknown routes to diagnosis were collapsed into a single ‘unknown and DCO’ category.

### Ethics

Data used in these analyses are collected and stored securely within NDRS under the legal permissions afforded by section 254 of the Health and Social Care Act 2012, which permits the collection of patient data without requiring informed consent.^17^^,^^18^ Ethical approval for genetic data analyses was granted to Can-Gene Can-Var (REC: 18/WS/0192). This analysis was approved by the NHS Digital analytical project panel (WP00481).

### Statistics

Descriptive analyses of the demographic and genetic characteristics of unique individuals in the ENLSR cohort were undertaken. We tested for equal distribution of MMR PV carriers across Indices of Multiple Deprivation (IMD) quintiles using the Chi-square Goodness of Fit test, for which all assumptions were satisfied. Crude prevalence rates of MMR PV carriers were calculated per Cancer Alliance per 100,000 population.

To avoid including patients for whom a cancer diagnosis had led to their diagnosis with LS, analyses comparing the ENLSR cohort to the general population, were limited to individuals who had received a predictive LS diagnosis. The number of unique individuals with cancer, and unique cancers in the ENLSR were counted. Median age at cancer diagnosis was calculated using only the first diagnosed cancer of that type for each individual. We assessed the distribution of cancer-specific age using visual inspection with QQ plots and the Shapiro–Wilk normality test, and found it not to be normally distributed. Thus, we performed statistical testing for a difference in medians between the ENLSR predictive population and the general population using the Wilcoxon Rank Sum test.

Distribution of route to diagnosis amongst cancers in the ENLSR predictive population and the general population was compared using the Chi-Squared test. Stage distribution of colorectal, endometrial, ovarian, upper GI, and urinary tract cancers combined in the ENLSR predictive population and the general population for the years 2016–2020 were compared using the Chi-Squared test. No multiplicity correction was applied to statistical analyses.

### Role of the funding source

The funder of this study had no role in study design, data collection, data analysis, data interpretation, or writing of the report.

## Results

### Data submission, extraction, and completeness

**Fig. 1:**
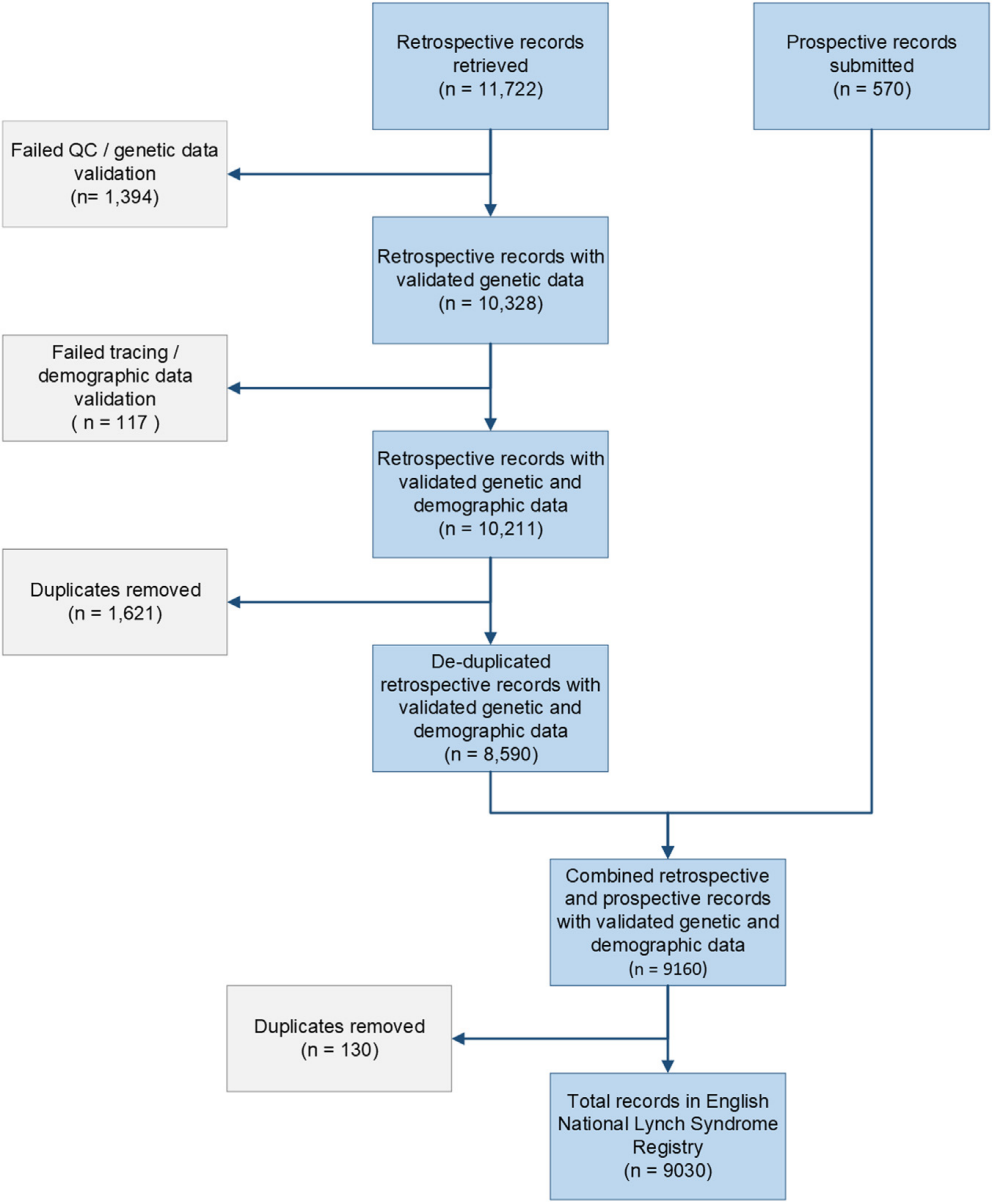
**Consort diagram showing flows of data into the English National Lynch Syndrome Registry.** Blue boxes show retained data, while grey boxes show data that was excluded, alongside the reason for exclusion. All counts are of data submissions.

The ENLSR is stored in a secure, queryable database held within NDRS. It contains all individuals identified by English NHS Regional Clinical Genetics Services as having a PV in at least one of the *MLH1*, *MSH2*, *MSH6*, *PMS2*, or *EPCAM* genes, who are traceable within the NHS Spine, and resident in England. A total of 11,722 retrospective submissions were received from 17 submitting centres (all Clinical Genetics Centres in England), of which 1394 failed genetic data validation, 117 failed demographic data validation, and 1621 were identified as duplicates, resulting in 8590 submissions for upload into the ENLSR database. As of July 2023, a total of 570 prospective submissions direct from genetics centres had passed automated genetic and demographic data validation, and were included in the ENLSR database, giving a total of 9160 records. Following de-duplication, 9030 records of unique individuals remained (Fig. 1). Data was >99% complete for all demographic variables, excepting ethnicity (Supplementary Table 1). For genetic variables, data completeness varied from 100.0% for LS gene, to 30.0% for LS variant pathogenicity class.

### Demographic and genetic characteristics

Of 9030 individuals in the ENLSR, 56.6% (n = 5112) are female and 43.4% (n = 3918) are male (Table 1). 43.7% (n = 3948) of MMR PV carriers are aged 50–74, 38.8% (n = 3507) are aged 25–49, 7.5% (n = 678) are aged 75–100, and 2.4% (n = 215) aged 0–24. 7.5% (n = 678) of MMR PV carriers in the ENLSR were deceased as of July 2023, with the remainder living (92.5%, n = 8348) or of unknown vital status (0.05%, n = 4). MMR PV carriers are not evenly distributed between IMD quintiles (Chi Squared, p < 0.0001), with a greater proportion of identified MMR PV carriers residing in less deprived areas (21.4% [n = 1930] of MMR PV carriers in IMD Quintile 5 [least deprived]) trending downwards to just 14.3% (n = 1291) in IMD Quintile 1 (most deprived).

**Table 1:** Demographic and genetic characteristics of the English National Lynch Syndrome Registry (ENLSR) cohort.

Variable	Category	Number	Percent (%)
Total MMR PV carriers		9030	
Demographic characteristics			
Gender	Male	3918	43.4
	Female	5112	56.6
Vital Status	Living	8348	92.5
	Deceased	678	7.5
	Unknown	4	0.05
Age	0–24	215	2.4
	25–49	3507	38.8
	50–74	3948	43.7
	75–100	678	7.5
	Deceased or unknown	682	7.6
Ethnicity	White	276	3.1
	Asian	18	0.2
	Black	10	0.1
	Chinese/Mixed/Other	15	0.2
	Unknown	8711	96.4
NHS Region	East of England	1014	11.2
	London	1172	13
	Midlands	1931	21.4
	North East and Yorkshire	1308	14.5
	North West	840	9.3
	South East	1414	15.7
	South West	1004	11.1
	Unknown	347	3.8
IMD Quintile	1 - most deprived	1291	14.3
	2	1708	18.9
	3	1854	20.5
	4	1900	21
	5 - least deprived	1930	21.4
	Unknown	347	3.8
Genetic characteristics			
Year of Lynch Syndrome diagnosis	1995–1999	65	0.7
	2000–2004	244	2.7
	2005–2009	720	8
	2010–2014	1201	13.3
	2015–2019	2520	27.9
	2020–2023	1959	21.7
	Unknown	2321	25.7
Age at Lynch Syndrome diagnosis	0–24	651	7.2
	25–49	3299	36.5
	50–74	2488	27.6
	75–100	271	3
	Unknown	2321	25.7
MMR gene	MLH1	2624	29.1
	MSH2	3362	37.2
	MSH6	1935	21.4
	PMS2	1109	12.3
Test scope	Diagnostic (LS diagnosis after first tumour diagnosis)	1981	21.9
	Predictive (LS diagnosis before first tumour diagnosis)	3239	35.9
	Unknown	3810	42.2

Includes 9030 MMR pathogenic variant carriers as of July 2023. Vital status, age and NHS Region are accurate as of July 2023. Test scope is determined by timing of Lynch Syndrome (LS) diagnosis relative to first cancer diagnosis. Tests are considered diagnostic if the LS diagnosis was made from 60 days before to any time after the first cancer diagnosis recorded for that individual. Tests are considered predictive if the LS diagnosis was made at least 60 days before the first cancer diagnosis recorded for that individual. EPCAM genes (n = 65) are included within the MSH2 category.

**Fig. 2:**
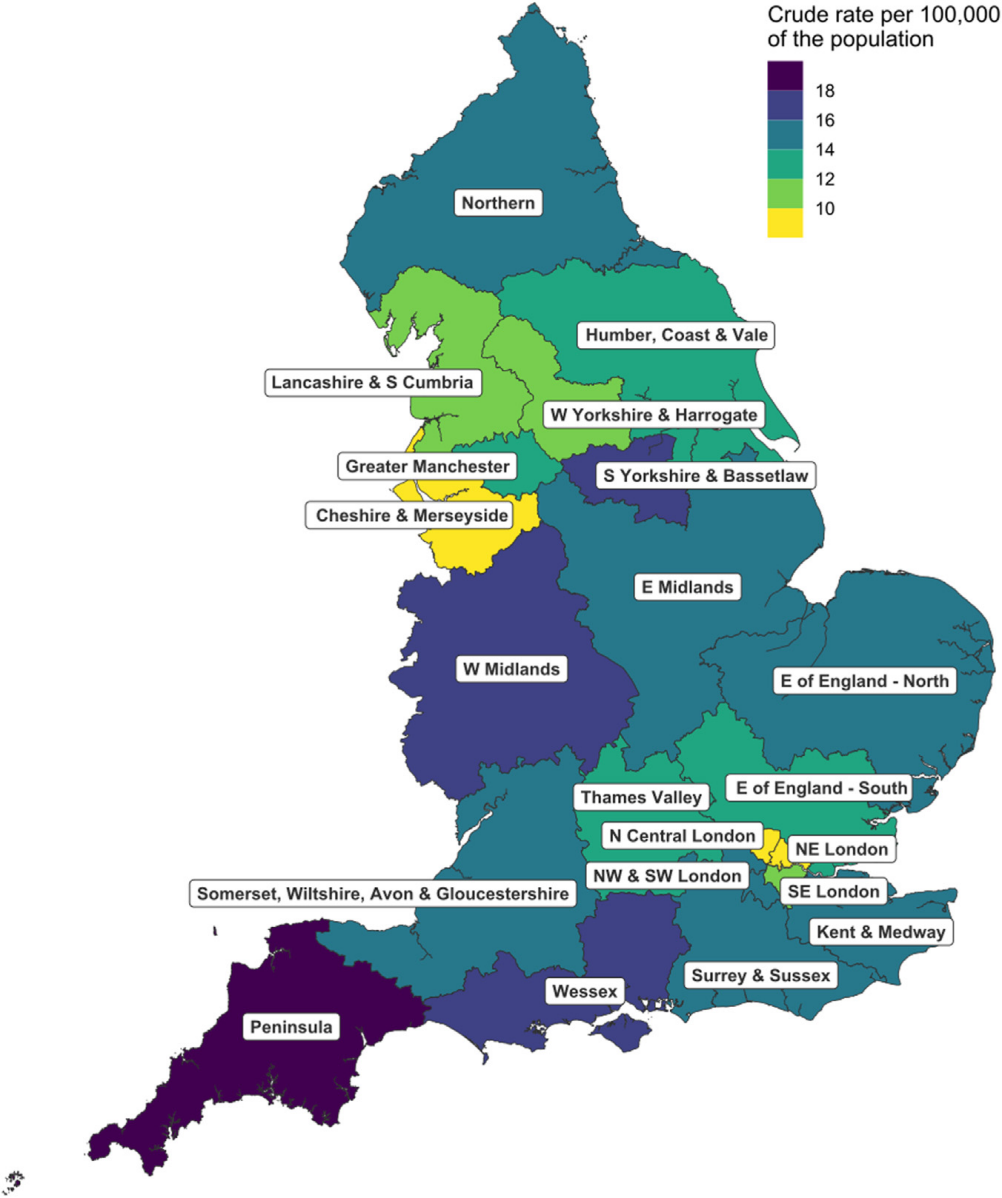
**Crude rate of MMR pathogenic variant carriers per 100,000 of the population by Cancer Alliance**.

Diagnosed MMR PV carriers are unevenly distributed across the country, with the crude rate of MMR PV carriers per 100,000 of the population ranging from 8.6 in the North East London Cancer Alliance to 18.8 in the Peninsula Cancer Alliance (Fig. 2, Supplementary Table 4).

*MSH2* and *MLH1* are the most frequently observed pathogenic MMR genes, at 37.2% (n = 3362) and 29.1% (n = 2624) of the ENLSR cohort, followed by 21.4% (n = 1935) and 12.3% (n = 1109) for *MSH6* and *PMS2* genes, respectively. The number of individuals diagnosed with LS increased over time, from just 65 individuals between 1995 and 1999, to 1959 between 2020 and 2023. Most (36.5%, n = 3299) individuals are diagnosed between the age of 25 and 49. For many MMR PV carriers, the relationship between LS being diagnosed and occurrence of first cancer remains unclear (42.2%, n = 3810), due to missing data on date of LS diagnosis or the date of LS diagnosis being after 2020 for which cancer registry data were unavailable. However, 35.9% (n = 3239) of MMR PV carriers in the ENLSR received their LS diagnosis before their first cancer diagnosis (presumptive predictive germline test) and 21.9% (n = 1981) are known to have been diagnosed with LS after their first cancer diagnosis (presumptive diagnostic germline test) (Table 1).

### Cancer diagnoses

41.4% (n = 3739) of the ENLSR cohort had at least one cancer diagnosis recorded in the NCRD between 1995 and 2020, comprising a total of 5262 tumours. The most frequently observed cancer is colorectal cancer (2942 tumours in 2519 MMR PV carriers), followed by endometrial cancer (836 tumours in 833 MMR PV carriers), Upper GI cancers (279 tumours in 265 MMR PV carriers) and urinary tract cancers (249 tumours in 215 MMR PV carriers) (Supplementary Table 5).

Restricting analysis to MMR PV carriers identified via presumptive predictive testing, 12.3% (n = 400) developed at least one cancer (prior to cancer data censor date of December 31, 2020). This compares to 35.6% (n = 1358) of recipients of germline MMR tests of unknown scope, and 100% (n = 1981) of recipients of diagnostic germline tests. The most frequently observed cancer in recipients of predictive germline tests is colorectal cancer, developed by 6.3% (n = 204) of MMR PV carriers, followed by endometrial (3.1%, n = 55), upper GI (1.4%, n = 44), urinary tract (1.1%, n = 36) and ovarian (0.5%, n = 8) (Table 2). The percent of presumptive predictive test recipients who developed at least one cancer also varied by MMR gene, at 15.7% (n = 161) of *MLH1* PV carriers, 14.7% (n = 183) of *MSH2* PV carriers, 7.4% (n = 47) of *MSH6* PV carriers, and 2.8% (n = 9) of *PMS2* PV carriers.

**Table 2:** Number of recipients of predictive germline MMR tests in the English National Lynch Syndrome Registry (ENLSR) cohort with a recorded tumour by MMR gene.

	Number of MMR PV Carriers			All cancers		Colorectal		Endometrial		Ovarian		Upper GI		Urinary tract	
MMR Gene	Male	Female	Total	N	%	N	%	N	%	N	%	N	%	N	%
MLH1	488	540	1028	161	15.7	97	9.4	18	3.3	4	0.7	12	1.2	13	1.3
MSH2	554	694	1248	183	14.7	94	7.5	23	3.3	4	0.6	25	2.0	20	1.6
MSH6	289	347	636	47	7.4	12	1.9	13	3.7	0	0.0	5	0.8	3	0.5
PMS2	131	196	327	9	2.8	1	0.3	1	0.5	0	0.0	2	0.6	0	0.0
Total^a^	1462	1777	3239	400	12.3	204	6.3	55	3.1	8	0.5	44	1.4	36	1.1

Germline MMR tests are considered predictive if the LS diagnosis was made at least 60 days before the first cancer diagnosis recorded for that individual. Only tumours diagnosed between 1995 and 2020 are included.

N = number, % = percent.

a Individuals with >1 cancer type may contribute to counts in >1 site category, but are only counted once in the All cancers category.

**Fig. 3:**
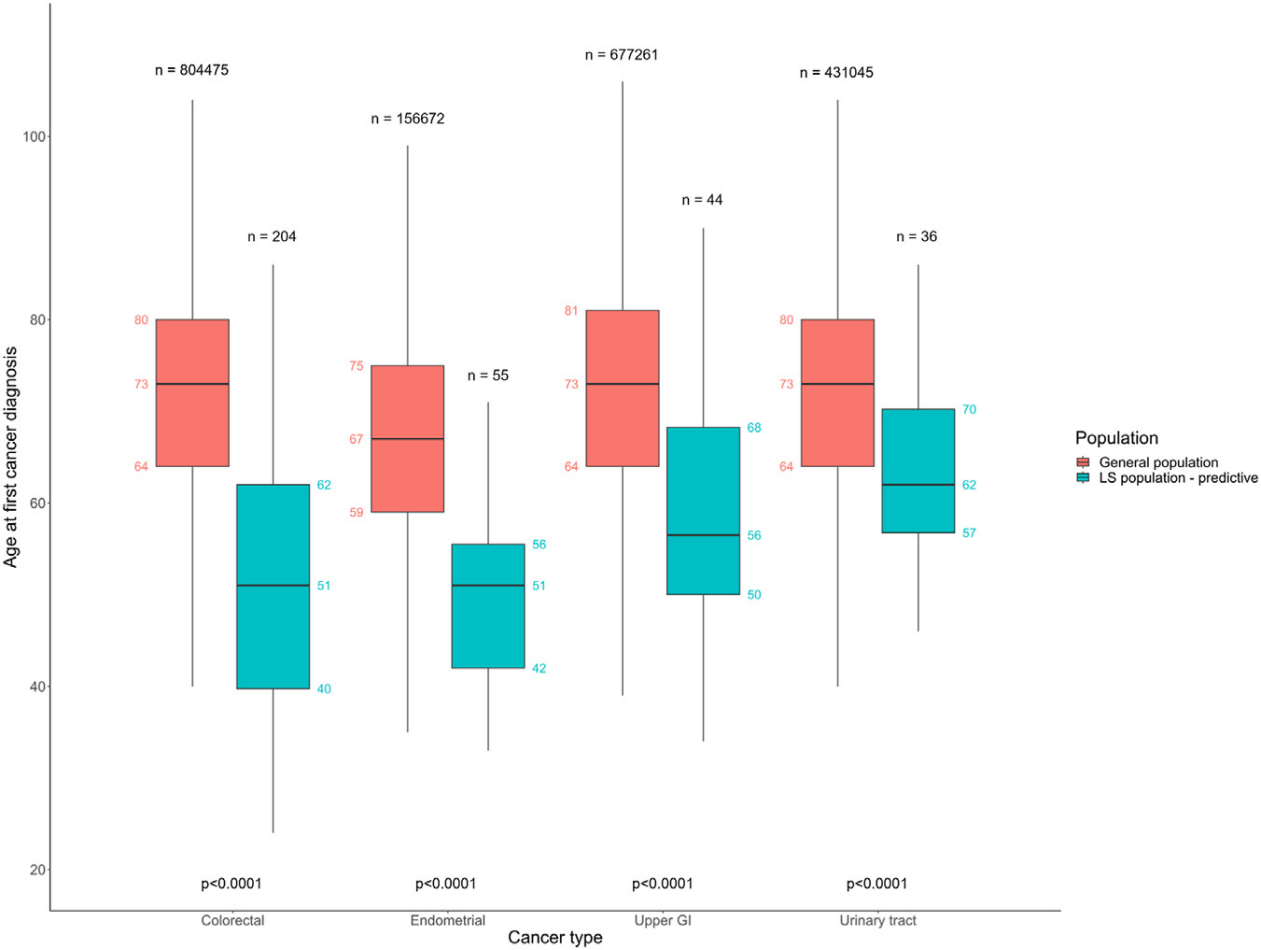
**Boxplot of age at diagnosis of first colorectal, endometrial, upper gastrointestinal (GI), and urinary tract cancers in the general population (orange) and in recipients of predictive germline mismatch repair (MMR) tests in the English National Lynch Syndrome Registry cohort (turquoise)**. Germline MMR tests are considered predictive if the Lynch Syndrome diagnosis was made at least 60 days before the first cancer diagnosis recorded for that individual. Only tumours diagnosed between 1995 and 2020 are included. Boxplots: lower and upper hinges correspond to the first and third quartiles respectively. Upper and lower whiskers extend to the largest or smallest value within 1.5 × the interquartile range from the upper and lower hinge respectively. n = sample size, p-values are the result of the Wilcoxon Rank Sum Test.

The median age at diagnosis of first colorectal, endometrial, ovarian, upper GI, and urinary tract cancers is lower in recipients of predictive germline MMR tests in the ENLSR population compared to the general population. In the general population, the median age at diagnosis of first colorectal cancer is 73 (IQR 64–80) compared to 51 (IQR 40–62) in ENLSR predictive test recipients (Wilcoxon Rank Sum, p < 0.0001). This pattern is also observed for endometrial cancer (general population median age 67 [IQR 59–75], ENLSR predictives median age 51 [IQR 42–56], p < 0.0001), ovarian cancer (general population median age 66 [IQR 55–76], ENLSR predictives median age 48 [IQR 47–53], p = 0.0041), upper GI cancer (general population median age 73 [IQR 64–81], ENLSR predictives median age 56 [IQR 50–68], p < 0.0001), and urinary tract cancer (general population median age 73 [IQR 64–80], ENLSR predictives median age 62 [IQR 57–70], p < 0.0001) (Fig. 3, Supplementary Table 8).

From 2006 to 2018, the routes to diagnosis of colorectal, endometrial, ovarian, upper GI, and urinary tract cancers in the ENLSR predictive population differed from that in the general population (Chi-squared, p < 0.0001). A smaller proportion of tumours in the ENLSR predictive population were diagnosed via the emergency route and a larger proportion by the routine route, which includes risk-based colonoscopic surveillance (Supplementary Table 6, Supplementary Fig. 2). Additionally, between 2016 and 2020, the stage distribution at diagnosis of colorectal, endometrial, ovarian, upper GI, and urinary tract cancers in the ENLSR predictive population differed from that in the general population (Chi-squared, p < 0.0001), with a larger proportion of tumours in the ENLSR predictive population diagnosed at an earlier stage (Supplementary Table 7, Supplementary Fig. 3).

## Discussion

We present here description of the ENLSR censored at July 25, 2023: a comprehensive, secure, national dataset comprising 9030 LS cases. This amalgamation of retrospectively and prospectively identified cases is complemented by development of infrastructure for prospective central registration of newly identified carriers, ensuring ongoing maintenance of a comprehensive national dataset.

The very modest number of MMR PV carriers identified nationally in the 27 years of NHS MMR genetic testing (1995–2023) is striking. Taking an estimate of prevalence of LS (MMR PVs) of 1 in 279,^19^ we would estimate there to be ∼175,000 MMR PV carriers in England, meaning we have identified less than 5% of MMR PV carriers.

In our data there is a predominance of *MSH2* PV carriers (37.2%, n = 3362), followed by *MLH1* (29.1%, n = 2624), *MSH6* (21.4%, n = 1935), *PMS2* (12.3%, n = 1109). This distribution differs from that of large-scale population cohorts; in UKBiobank for example the reported distribution of Lynch PVs is: 7.9% (n = 6) *MSH2* PV carriers, 25.0% (n = 19) *MLH1* PV carriers, 56.6% (n = 43) *MSH6* PV carriers, and 10.5% (n = 8) *PMS2* PV carriers.^20^ Thus, the registry distribution indicates that, although still low percentages of the projected national MMR PV totalities, our ascertainment of PV-carriers is proportionately greater for the higher penetrance genes and lower for *PMS2* and *MSH6*. The distribution in the registry reflects more testing for *MLH1* and *MSH2*, due to i) the earlier discovery of these genes and ii) technical challenges due to pseudogenes delaying widespread testing of *PMS2.*^21^ It also likely reflects the high historical threshold for testing, which corresponded to a series in which enrichment for high penetrance genes would be anticipated. As we test more broadly (including potentially via population-based programs), the proportion will increase for *PMS2* PVs, which are more common but of lower penetrance.

The established cancer risks differ between *MLH1*, *MSH2*, *MSH6*, and *PMS2* and this is reflected in the cancer incidences for PV carriers for the four genes, although this may also in part reflect ascertainment bias due to eligibility criteria for testing. Indeed, early estimates of risk were highly biased by ascertainment on the basis of associated cancers. These estimates have been improved with prospective follow-up data from unaffected individuals ascertained via familial (predictive) testing. However, aggressive screening, early polypectomy, aspirin chemoprophylaxis, and preventative gynaecological surgery will all now influence penetrance estimates. Consistent with early identification of the genes via linkage analysis, *MLH1* and *MSH2* confer higher cancer risks overall, especially for colorectal cancer. PVs in *MSH6* confer a high risk of endometrial cancer but modest risk of colorectal cancer. The risks of all cancers for *PMS2* PV carriers are especially modest, in particular for colorectal cancer.^9^ It is an accepted limitation that the pattern of cancer incidence across the MMR PV carriers in the ENLSR is influenced by case ascertainment including changes in patterns of gene testing and test eligibility criteria over time.

It is noteworthy that female MMR PV carriers outnumber males: this may in part reflect greater health-seeking behaviour of women in regard of pursuing genetic testing for which they are eligible. Whilst there will also be a bias towards female eligibility from personal diagnosis with endometrial cancer, this is likely counter-balanced by the higher age-adjusted incidence of colorectal and other gastrointestinal cancers in males.

The age distribution of MMR PV carriers will be influenced by multiple factors relating to ascertainment, cancer incidence and population structure. However, that the bulk of living MMR PV-carriers are aged 50–74 is important as this is the age of highest absolute underlying risk of colorectal and endometrial cancer, underscoring the importance of systematic oversight of interventions for surveillance, surgical risk reduction, and aspirin chemoprevention in this group. There is marked variation in the geographical distribution of MMR PV carriers; whilst this is indicative of clinical variability in genetic testing, more detailed evaluation is required with regard to regional population age structure, cancer incidence, impact of Scottish and Welsh borders and record-keeping at specific locations.

Likewise, preliminary evaluation of the pattern of cancer diagnosis indicates, as expected, that cancers diagnosed in MMR PV-carriers are at a younger age that in the broader population. Also, an indicator of efficacy of screening in LS, the cancers are more likely to be diagnosed at an earlier stage and are more likely to be diagnosed via routine follow-up (including regular surveillance colonoscopy) than as an emergency or as an urgent symptomatic presentation.

There were a number of limitations inherent to the ascertainment process for retrospective cases and their related data. Through the mandate of a formal national NHS-led GMSA initiative, all clinical centres and all laboratories who have ever undertaken MMR genetic analyses were required to submit accrued historic data against a prescribed data model. Nevertheless, it is not possible to guarantee full totality of cases nationally.

Furthermore, the extent of completeness of submission against the data model also presents limitations. While demographic details were generally well-populated, genetic data was missing for several individuals. In particular, missingness on data regarding date of LS diagnosis prevents adjustment for ascertainment, and limits time-course analyses of disease penetrance as well as evaluations of temporal patterns of genetic testing in relation to implementation of NICE guidance or the COVID-19 pandemic. Missingness of variant nomenclature details precludes evaluation of variant pathogenicity classifications. It is anticipated that some of these missing data elements may be retrievable by linkage with an existing national dataset of MMR genomic laboratory records in NDRS in the future, following the harmonisation of patient identifiers across both datasets.^21^

Whilst linkage to the NCRD offers opportunity for systematic assessment of incidence of cancers pre-and post-ascertainment of the LS pathogenic variant, the dataset has some limitations in regard of (i) registration of cancers only being robustly linked to NHS Number subsequent to 1995 (ii) pre-invasive lesions not being captured (iii) only tumours diagnosed within the English NHS being registered. Due to lag times inherent to the cancer registration processes, cancer incidence is complete to December 2020 only.

Furthermore, whilst our analyses offer some preliminary glimpses at geographic patterns of ascertainment of LS carriers, temporal analyses adjusted for the underlying population age and gender structure are required for formal evaluation of patterns of LS testing.

Thus, albeit with limitations in regard of some elements of data completeness, the ENLSR represents a comprehensive national registry of MMR PV carriers and one of the largest series of MMR PV carriers worldwide. Furthermore, by virtue of the governance structure, relational database, and presence of unique linkable identifier, the registry can readily be linked to many other datasets, including hospital episodes statistics (HES), Office of National Statistics (ONS) vital status data, prescribing data, and the multiple NCRAS datasets, including the Systemic Anti-Cancer Therapy dataset (SACT) and National Radiotherapy Dataset (RTDS), enabling the investigation of management and outcomes.^22^^,^^23^ In addition, on account of creation of a simple portal for real-time submission to the registry of newly ascertained MMR PV-carriers and mandated participation, the ENLSR will continue to grow. The registry will support systematic centralised national delivery of colonoscopy via the National Bowel Screening Programme, as well as longitudinal evaluation of management practices and impact. Importantly, the ENLSR is also currently the only registry of PV carriers in England. Hence, the ENLSR might serve as a blueprint for national registries of PV carriers of other genes, in particular those relating to cancer susceptibility for whom regular surveillance is indicated.

The amalgamation of historic MMR PV carriers has represented a significant challenge; lessons from this as well as the development of the data portal for prospectively ascertained MMR PV-carriers will be instructive for any rollout into other groups. Alongside the technological hurdles in introduction of genomics into healthcare, are these more prosaic hurdles of data collation and administration. Detailed attention is required in the assembly and integration of these data to ensure the benefits of genomic information are genuinely realised.

## Contributors

J.B. and C.T. obtained research funding supporting analyses. C.H. and C.T. designed the analyses. C.H. coded the analyses and L.L. quality assured the analyses. Both C.H. and L.L. accessed and verified the underlying data and generated tables and figures for presentation. S.H., A.S., K.M. and J.B. designed and oversaw the establishment of the LS Registry, with support from C.M., R.B., and C.H. R.B. and C.M. coordinated LS data retrieval. O.T., X.Z., J.L., P.E., and N.A. built the online submission portal for prospective data submissions. R.B., S.H., C.M. and F.M. quality assured the LS data. C.M. provided LS data for analysis. T.R. performed linkage of LS data to the cancer registry. B.T. provided project management for data access and analyses. C.T. and E.M. provided clinical interpretations of data. C.H., L.L. and C.T. drafted the manuscript. All authors read and approved the final version of the manuscript.

## Data sharing statement

Summary data relevant to the study are included in the article or supplementary information. Individual-level data used in this study are held within NDRS under robust privacy, security and confidentiality procedures and only available to properly authorised analysts and researchers under access arrangements through the NDRS Data Access and Release Service.

## Declaration of interests

C.T. has received personal fees from Astra Zeneca and Roche. K.M. acts as a medical advisor for Bowel Cancer UK, Lynch Syndrome UK, and NHS England.

## Appendix A Supplementary data

Supplementary Methods, Tables 1–8 and Figures 1–3

GMSA Lynch Consortium Author List
