# Supplementary material for: The comprehensive English National Lynch Syndrome Registry: development and description of a new genomics data resource
Source: eClinicalMedicine. 2024 Feb 7;69:102465. doi: 10.1016/j.eclinm.2024.102465 (PMC10864212; doi:10.1016/j.eclinm.2024.102465)
Supplement: Supplementary Methods, Tables 1–8 and Figures 1–3 [file mmc1.docx]

**The comprehensive English National Lynch Syndrome Registry: development and description of a new genomics data resource**

**Supplementary materials**

Table of Contents

[Supplementary methods 2](#_Toc152258702)

[Establishment of the English National Lynch Syndrome Registry 2](#_Toc152258703)

[Analysis of the English National Lynch Syndrome Registry and linked cancer data 3](#_Toc152258704)

[Supplementary tables 5](#_Toc152258705)

[Supplementary Table 1 - Common data model for the English National Lynch Syndrome Registry 5](#_Toc152258706)

[Supplementary Table 2 - Data conflicts and rules for selecting preferred records 6](#_Toc152258707)

[Supplementary Table 3 - Sources of data used in analyses 7](#_Toc152258708)

[Supplementary Table 4 - Crude rate of MMR Pathogenic Variant Carriers per 100,000 of the population in each Cancer Alliance 8](#_Toc152258709)

[Supplementary Table 5 - Cancers in the Lynch Syndrome Registry cohort 9](#_Toc152258710)

[Supplementary Table 6 - Route to diagnosis of colorectal, endometrial, ovarian, upper GI, and urinary tract tumours in the general population and in recipients of predictive germline MMR testing in the English National Lynch Syndrome Registry (ENLSR) 10](#_Toc152258711)

[Supplementary Table 7 - Stage at diagnosis of colorectal, endometrial, ovarian, upper GI, and urinary tract tumours in the general population and recipients of predictive germline MMR tests in the English National Lynch Syndrome Registry (ENLSR) 2016-2020 11](#_Toc152258712)

[Supplementary Table 8 - Median age at diagnosis of first colorectal, endometrial, ovarian, upper GI, and urinary tract tumours in the general population and recipients of predictive germline MMR tests in the English National Lynch Syndrome Registry (ENLSR) cohort. 12](#_Toc152258713)

[Supplementary figures 13](#_Toc152258714)

[Supplementary figure 1 – overview of methods and processes used in analyses 13](#_Toc152258715)

[Supplementary Figure 2 – Route to diagnosis of colorectal, endometrial, ovarian, upper GI, and urinary tract tumours in the general population and in recipients of predictive germline MMR testing in the English National Lynch Syndrome Registry (ENLSR) 14](#_Toc152258716)

[Supplementary Figure 3 - Stage at diagnosis of colorectal, endometrial, ovarian, upper GI, and urinary tract tumours in the general population and recipients of predictive germline MMR tests in the English National Lynch Syndrome Registry (ENLSR) 2016-2020 15](#_Toc152258717)

[References 16](#_Toc152258718)

## Supplementary methods

### Establishment of the English National Lynch Syndrome Registry

Automated data extraction pilot: The piloted system of automated data extraction, restructuring, and amalgamation consisted of using Regular Expressions identification using R (v4·3·1) to extract key information from free text to populate the common data model. This process was replaced by a manual process due to erroneous and missing data items identified on automated extraction.

Manual data extraction and quality control: We assembled a panel of experts with genetics training, who reviewed individual submissions (line by line) to i) extract genetic data from free text, and ii) conduct quality control (identification of submissions with insufficient data, inconsistent data, or containing errors). Each individual submission was independently assessed by at least two trained reviewers, and the results of these assessments compared. Where both reviewers agreed on data extraction and noted no quality control issues, the submission was considered to have passed extraction and quality control and progressed to the next stage. Where different results were obtained by each reviewer in the data extraction or quality control assessment, the submission was flagged. Flagged submissions were discussed by the two independent reviewers until agreement was reached, with referral to a third arbiter if required. If both assessors determined that a quality control issue was present, the submission was considered to have failed genetic data validation and was returned to the source of the original submission, alongside a request for clarification.

### Analysis of the English National Lynch Syndrome Registry and linked cancer data

Statistical software: All analyses were performed in R (v4·3·1) using the *Tidyverse (v2.0.0)*^2^*, ggpubr (v0.6.0)t*^3^*, svglite (v2.1.2)*^4^*, sf (v1.0.14)*^5^*,* and *viridis (v0.6.4)*^6^ packages. A summary of all data sources used in this analysis is provided in Supplementary Table 3.

Handling of erroneous LS diagnosis dates: We assumed that any dates of Lynch Syndrome diagnosis that matched the individual’s date of birth (n=6) were erroneous set them to null (five had dates of diagnosis prior to 1995, and one between 1995 and 2000).

Cancer case counting procedures: We extracted data on all cancers occurring between January 1, 1995, and December 31, 2020, excluding tumours diagnosed outside England, tumours with suspected incorrect age at diagnosis (outside of the range 0-200), tumours assigned to individuals of unknown gender, tumours where gender is incompatible to tumour (females with site codes in the range C60-C63, or males with site codes in the range C51 to C58), non-invasive tumours (ICD-10 codes beginning with D), and non-melanoma skin cancers (ICD-10 code C44).

## Supplementary tables

Supplementary Table 1 - Common data model for the English National Lynch Syndrome Registry. Variables listed are those included in the ENLSR, their definition, and completeness for the registry censored at July 2023. LS = Lynch Syndrome. NDRS = National Disease Registration Service.

| **Variable** | **Definition** | **Data completeness (%)** |
| --- | --- | --- |
| Provider organisation code | Unique code assigned to centre submitting data | 100 |
| NDRS local ID | Unique code assigned to individual record within NDRS | 99.9 |
| First name | First name of MMR pathogenic variant carrier | 100·0 |
| Last name | Last name of MMR pathogenic variant carrier | 100·0 |
| Date of birth | Date of birth of MMR pathogenic variant carrier | 100·0 |
| Vital status | Vital status of MMR pathogenic variant carrier | 0·04 |
| Date of death | Date of death of MMR pathogenic variant carrier | 0·04 |
| Gender | Gender of MMR pathogenic variant carrier | 100·0 |
| Ethnicity | Self-reported ethnicity of MMR pathogenic variant carrier | 3·5 |
| Postcode | Current postcode of residence of MMR pathogenic variant carrier | 99·6 |
| NHS number | NHS number of MMR pathogenic variant carrier | 100·0 |
| Date of diagnosis | Best estimate of date of diagnosis with Lynch Syndrome | 74·3 |
| LS gene | MMR gene in which the pathogenic variant has been identified | 100·0 |
| LS variant | Pathogenic variant identified in MMR gene | 53·2 |
| LS variant pathogenicity class | Pathogenicity class of the MMR variant (as assessed by diagnosing centre at the time of diagnosis) | 30·0 |

Supplementary Table 2 - Data conflicts and rules for selecting preferred records. A data conflict occurs when different information relating to the same MMR PV carrier (as identified by shared NHS Number and DOB) is submitted on different dates or by different organisations. Where this occurs, the most recent (prospective) data is preferentially selected, as it likely constitutes an update to historical (retrospective) data. If more than one prospective submission is identified, variable-specific rules are used to select the preferred record. First name and last name were not identifiers used in this analysis owing to the potential for variations in spelling or typographical errors.

| **Variable** | **Individuals (n) with identified data conflicts** | **Rule for selecting preferred record** |
| --- | --- | --- |
| Provider organisation code | 22 | Prospective data preferentially selected, set to null if no/conflicting prospective data |
| First name | 16 | N/A - not key identifier |
| Last name | 5 | N/A - not key identifier |
| Date of birth | 0 | N/A - no conflicts identified |
| Vital status | 0 | N/A - no conflicts identified |
| Date of death | 0 | N/A - no conflicts identified |
| Gender | 0 | N/A - no conflicts identified |
| Ethnicity | 0 | N/A - no conflicts identified |
| Postcode | 8 | Prospective data preferentially selected, set to null if no/conflicting prospective data |
| NHS number | 0 | N/A - no conflicts identified |
| Date of diagnosis | 40 | Earliest date of diagnosis selected |
| LS gene | 0 | N/A - no conflicts identified |
| LS variant | 59 | Prospective data preferentially selected; set to null if no/conflicting prospective data |
| LS pathogenicity class | 3 | Prospective data preferentially selected; set to null if no/conflicting prospective data |

Supplementary Table 3 - Sources of data used in analyses. Includes headline description of data and period covered, as well as dataset’s host organisation.

| **Data** | **Details** | **Reference / Source** |
| --- | --- | --- |
| Demographic and genetic data for MMR PV carriers | Demographic and genetic data from all known MMR pathogenic variant carriers in England | National Lynch Syndrome Registry, National Disease Registration Service (NDRS) |
| Cancer data for LS Registry cohort | ICD-10 codes, date of diagnosis, stage at diagnosis, and route to diagnosis of tumours diagnosed in members of the Lynch Registry cohort in England between 1995 and 2020. | National Cancer Registration Dataset, National Cancer Registration and Analysis Service (NCRAS), NDRS^10,11^ |
| Cancer data for general population | ICD-10 codes, date of diagnosis, stage at diagnosis, and route to diagnosis of tumours diagnosed in the general population (members of the Lynch Registry cohort excluded) in England between 1995 and 2020. | National Cancer Registry, NCRAS, NDRS^11,12^ |
| Geography data | Lookup tables mapping postcodes to LSOA, cancer alliance, integrated care board and NHS Region. | NCRAS, NDRS^11,12^ |
| IMD Quintiles | Lookup tables mapping LSOA to Index of Multiple Deprivation (IMD) Quintiles | NCRAS, NDRS^11,12^ |
| Cancer alliance population sizes | Population sizes for each Cancer Alliance in 2020 | NCRAS, NDRS^11,12^ |
| Geographical boundaries | Shapefile containing geographical boundaries of Cancer Alliances (meeting 2020 definitions) in England | ONS Open Geography Portal^13^ |
| Demographic details for MMR pathogenic variant carriers | Source of up-to-date demographic data for NHS-registered individuals in England, used to validate and update demographic data from the English National Lynch Syndrome Registry | NHS Spine, NHS England^1^ |

Supplementary Table 4 - Crude rate of MMR Pathogenic Variant Carriers per 100,000 of the population in each Cancer Alliance. Number of MMR PV carriers derived from the Lynch Syndrome Registry Cohort (as of July 2023), and matched to Cancer Alliance by postcode of residence. Population sizes derived from NCRAS population data for 2020.

| **Cancer Alliance** | **Number of MMR PV carriers** | **Crude rate of MMR PV carriers (per 100,000 of the population)** |
| --- | --- | --- |
| Cheshire and Merseyside | 218 | 8.7 |
| East Midlands | 738 | 15.7 |
| East of England North | 449 | 15.4 |
| East of England South | 506 | 13.9 |
| Greater Manchester | 373 | 12.9 |
| Humber, Coast and Vale | 230 | 13.5 |
| Kent and Medway | 268 | 14.3 |
| Lancashire and South Cumbria | 205 | 12 |
| North Central London | 152 | 10 |
| North East London | 175 | 8.6 |
| Northern | 457 | 15.2 |
| Peninsula | 335 | 18.8 |
| RM Partners West London | 576 | 15.9 |
| Somerset, Wiltshire, Avon and Gloucestershire | 437 | 14.1 |
| South East London | 219 | 12 |
| South Yorkshire and Bassetlaw | 247 | 16.1 |
| Surrey and Sussex | 554 | 15.8 |
| Thames Valley | 222 | 12.9 |
| Unknown | 316 | 10 |
| Wessex | 428 | 16.4 |
| West Midlands | 959 | 16.1 |
| West Yorkshire and Harrogate | 284 | 11.9 |

Supplementary Table 5 - Cancers in the Lynch Syndrome Registry cohort. Total number of tumours of each type, as well as people with at least one tumour of each type, in the full Lynch Syndrome Registry cohort, and according to test scope. Test scope is determined by timing of Lynch Syndrome (LS) diagnosis relative to first cancer diagnosis. Tests are considered diagnostic if the LS diagnosis was made from 60 days before to any time after the first cancer diagnosis recorded for that individual (60-day window chosen to allow for inaccuracies in LS diagnostic dates). Tests are considered predictive if the LS diagnosis was made at least 60 days before the first cancer diagnosis recorded for that individual. A test scope is unknown if the date of LS diagnosis is unknown or took place after 31-12-2020 (as cancer diagnosis data is not available after this date). *Totals for counts of people do not sum as people may be included in more than one category, for example if an individual has both a colorectal and an endometrial cancer, they will be counted once in each row.

|  |  | **Full cohort**  **(n = 9030)** | | **Diagnostic germline MMR test (n = 1981)** | | **Predictive germline MMR test (n = 3239)** | | **Scope of germline MMR test unknown**  **(n = 3810)** | |
| --- | --- | --- | --- | --- | --- | --- | --- | --- | --- |
| **Cancer type** | **ICD-10 Codes** | **People  (n)** | **Tumours**  **(n)** | **People  (n)** | **Tumours**  **(n)** | **People  (n)** | **Tumours**  **(n)** | **People  (n)** | **Tumours**  **(n)** |
| Colorectal | C18, C19, C20 | 2519 | 2942 | 1429 | 1708 | 204 | 225 | 886 | 1009 |
| Endometrial | C54 | 833 | 836 | 468 | 469 | 55 | 56 | 310 | 311 |
| Urinary tract | C64, C65, C66, C67, C68 | 215 | 249 | 110 | 135 | 36 | 37 | 69 | 77 |
| Ovarian | C48, C56, C57 | 187 | 194 | 112 | 116 | 8 | 8 | 67 | 70 |
| Upper GI | C15, C16, C17, C22, C23, C24, C25 | 265 | 279 | 120 | 128 | 44 | 47 | 101 | 104 |
| Other | All other codes excluding C44 | 703 | 762 | 338 | 376 | 104 | 111 | 261 | 275 |
| Total* |  | 3739 | 5262 | 1981 | 2932 | 400 | 484 | 1358 | 1846 |

Supplementary Table 6 - Route to diagnosis of colorectal, endometrial, ovarian, upper GI, and urinary tract tumours in the general population and in recipients of predictive germline MMR testing in the English National Lynch Syndrome Registry (ENLSR)**.** Only tumours diagnosed between 2006 and 2018 included (route to diagnosis not available for other years). Germline MMR tests are considered predictive if the LS diagnosis was made at least 60 days before the first cancer diagnosis recorded for that individual. Routes to diagnosis include emergency, Routine (GP referral, inpatient elective, other outpatient), Screening, Two-week wait, or unknown or death certificate only. Cancers diagnosed via screening in recipients of presumptive predictive tests in the ENLSR are included in the routine category.

| **Cancer type** | **Route to diagnosis** | **General population** | **Recipients of predictive germline MMR tests in the ENLSR** |
| --- | --- | --- | --- |
|  |  | Percent of total (%) | Percent of total (%) |
| Colorectal, Endometrial, Ovarian, Upper GI, Urinary tract | Emergency | 25·7 | 8·1 |
|  | Routine | 38·1 | 75·3 |
|  | Screening | 2·8 | - |
|  | Two-week wait | 29·7 | 13·7 |
|  | Unknown or death certificate only (DCO) | 3·7 | 3·0 |

Supplementary Table 7 - Stage at diagnosis of colorectal, endometrial, ovarian, upper GI, and urinary tract tumours in the general population and recipients of predictive germline MMR tests in the English National Lynch Syndrome Registry (ENLSR) 2016-2020**.** Only tumours diagnosed in the past 5 years are included (2016-2020). Germline MMR tests are considered predictive if the LS diagnosis was made at least 60 days before the first cancer diagnosis recorded for that individual.

| **Cancer type** | **Stage at diagnosis** | **General population** | **Recipients of predictive germline MMR tests in the ENLSR** |
| --- | --- | --- | --- |
|  |  | Percent of total (%) | Percent of total (%) |
| Colorectal, Endometrial, Ovarian, Upper GI, Urinary tract | Stage 1 | 22·2 | 44·7 |
|  | Stage 2 | 14·5 | 23·1 |
|  | Stage 3 | 19·6 | 16·8 |
|  | Stage 4 | 24·2 | 3·8 |
|  | Unknown | 19.4 | 11·5 |

### Supplementary Table 8 - Median age at diagnosis of first colorectal, endometrial, ovarian, upper GI, and urinary tract tumours in the general population and recipients of predictive germline MMR tests in the English National Lynch Syndrome Registry (ENLSR) cohort.

Germline MMR tests are considered predictive if the LS diagnosis was made at least 60 days before the first cancer diagnosis recorded for that individual. Only tumours diagnosed between 1995-2020 are included. *p-values are the result of the Wilcoxon Rank Sum Test

|  | **General population** | | **Recipients of predictive germline MMR tests in the Lynch Syndrome Registry cohort** | |  |
| --- | --- | --- | --- | --- | --- |
| **Cancer type** | **Median** | **(IQR)** | **Median** | **(IQR)** | **p-value*** |
| Colorectal | 73 | (64-80) | 51 | (40-62) | <0·0001 |
| Endometrial | 67 | (59-75) | 51 | (42-56) | <0·0001 |
| Ovarian | 66 | (55-76) | 48 | (47-53) | 0·0041 |
| Upper GI | 73 | (64-81) | 56 | (50-68) | <0·0001 |
| Urinary tract | 73 | (64-80) | 62 | (57-70) | <0·0001 |

## Supplementary figures

Supplementary figure 1 – overview of methods and processes used in analyses**.** Processes used for retrospective data (collected before January 1, 2023) are shown on the left side of the diagram, with those for prospective data (collected between January 1, 2023 and July 25, 2023) shown on the right side. Processes in the midline were applied to both prospective and retrospective data.


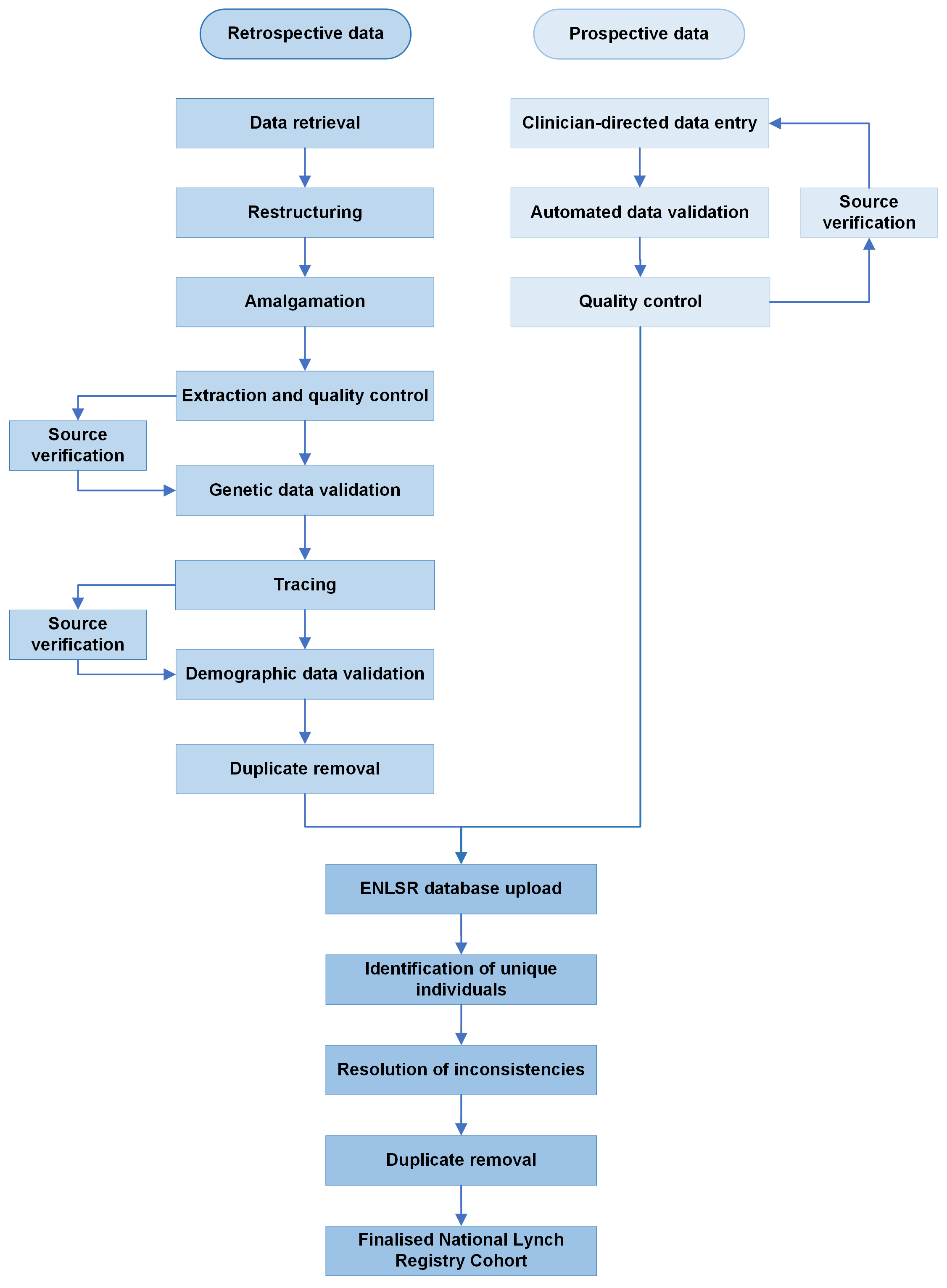


Supplementary Figure 2 – Route to diagnosis of colorectal, endometrial, ovarian, upper GI, and urinary tract tumours in the general population and in recipients of predictive germline MMR testing in the English National Lynch Syndrome Registry (ENLSR)**.** Only tumours diagnosed between 2006 and 2018 included (route to diagnosis not available for other years). Germline MMR tests are considered predictive if the LS diagnosis was made at least 60 days before the first cancer diagnosis recorded for that individual. Routes to diagnosis include Emergency, Routine (GP referral, inpatient elective, other outpatient), Screening, Two-week wait, or unknown or death certificate only. Cancers diagnosed via screening in recipients of presumptive predictive tests in the ENLSR are included in the routine category.

**
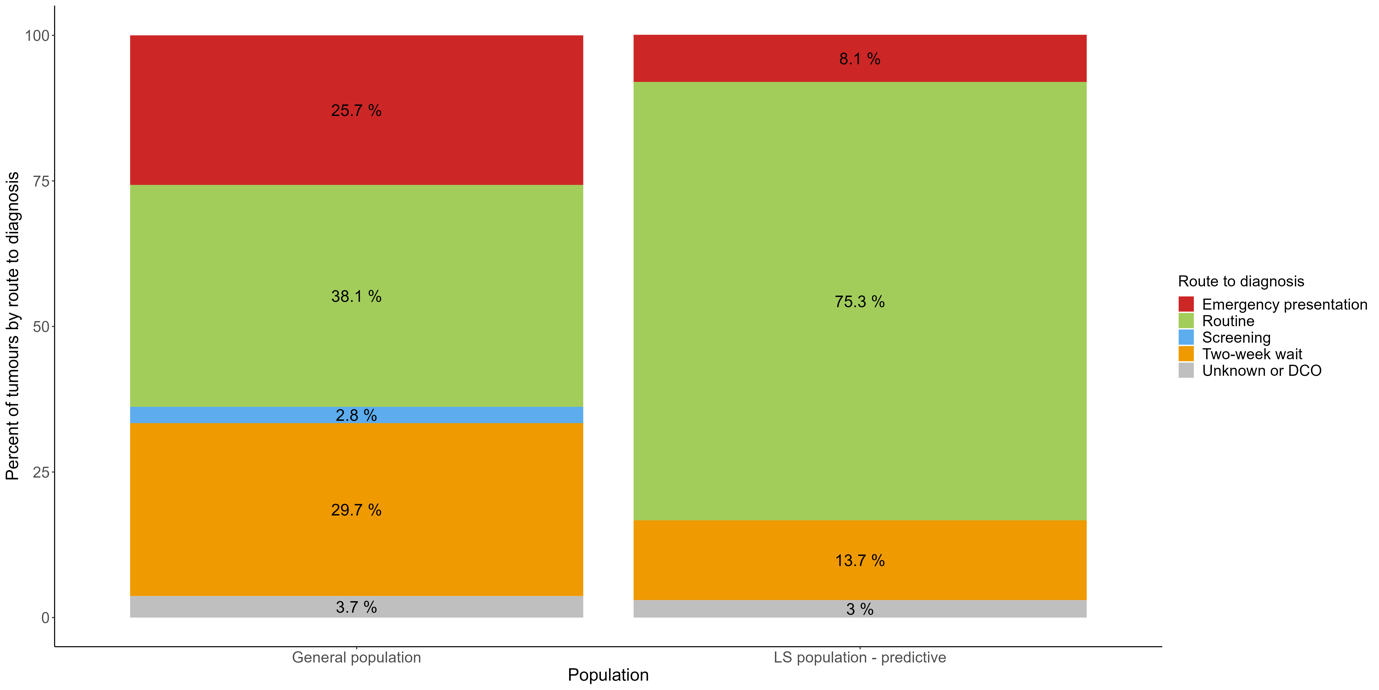
**

Supplementary Figure 3 - Stage at diagnosis of colorectal, endometrial, ovarian, upper GI, and urinary tract tumours in the general population and recipients of predictive germline MMR tests in the English National Lynch Syndrome Registry (ENLSR) 2016-2020. Only tumours diagnosed in the past 5 years are included (2016-2020). Germline MMR tests are considered predictive if the LS diagnosis was made at least 60 days before the first cancer diagnosis recorded for that individual.


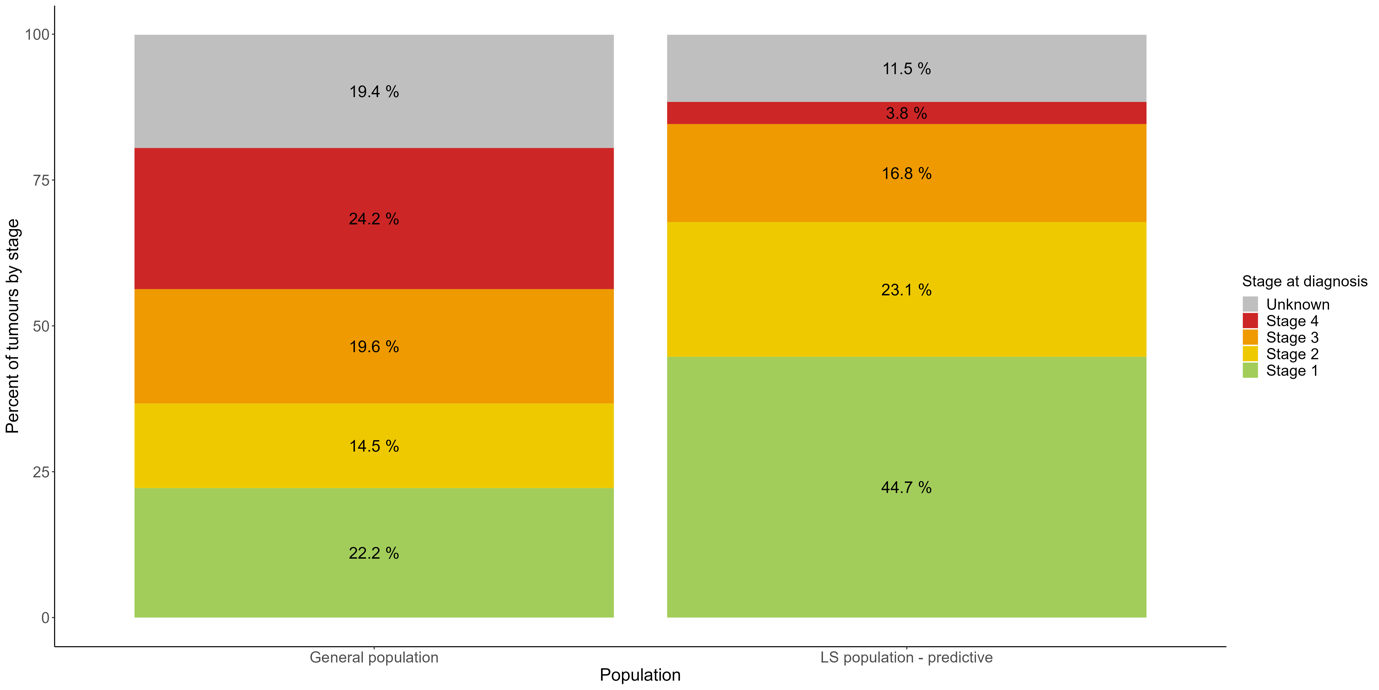


## References

1. Spine [Internet]. NHS Digital. [cited 2023 Aug 9]. Available from: https://digital.nhs.uk/services/spine

2. Wickham H, Averick M, Bryan J, Chang W, McGowan LD, François R, et al. Welcome to the Tidyverse. J Open Source Softw. 2019 Nov 21;4(43):1686.

3. Kassambara A. ggpubr: ‘ggplot2’ Based Publication Ready Plots [Internet]. 2023 [cited 2023 Nov 21]. Available from: https://rpkgs.datanovia.com/ggpubr/authors.html#citation

4. Wickham H, Henry L, Pedersen TL, Luciani TJ, Decorde M, Vaudor L. svglite: An ‘SVG’ Graphics Device [Internet]. 2023. Available from: https://svglite.r-lib.org, https://github.com/r-lib/svglite

5. Pebesma E. Simple Features for R: Standardized Support for Spatial Vector Data. R J. 2018;10(1):439–46.

6. Garnier S, Ross N, Rudis boB, Filipovic-Pierucci A, Galili T, timelyportfolio, et al. sjmgarnier/viridis: CRAN release v0.6.3 [Internet]. Zenodo; 2023 [cited 2023 Oct 2]. Available from: https://zenodo.org/record/7890878

7. Cancer Alliances (April 2020) Full Clipped Boundaries EN [Internet]. [cited 2023 Sep 15]. Available from: https://geoportal.statistics.gov.uk/datasets/ons::cancer-alliances-april-2020-full-clipped-boundaries-en/explore

8. NHS Digital. Routes to Diagnosis: Background [Internet]. NHS Digital. [cited 2023 Sep 27]. Available from: https://digital.nhs.uk/data-and-information/publications/statistical/routes-to-diagnosis/2018/background

9. Elliss-Brookes L, McPhail S, Ives A, Greenslade M, Shelton J, Hiom S, et al. Routes to diagnosis for cancer – determining the patient journey using multiple routine data sets. Br J Cancer. 2012 Oct 9;107(8):1220–6.

10. Henson KE, Brock R, Shand B, Coupland VH, Elliss-Brookes L, Lyratzopoulos G, et al. Cohort profile: prescriptions dispensed in the community linked to the national cancer registry in England. BMJ Open. 2018 Jul 1;8(7):e020980.

11. NDRS Cancer Registration [Internet]. NHS Digital. [cited 2023 Oct 2]. Available from: https://digital.nhs.uk/services/data-access-request-service-dars/dars-products-and-services/data-set-catalogue/ndrs-cancer-registration

12. Henson KE, Elliss-Brookes L, Coupland VH, Payne E, Vernon S, Rous B, et al. Data Resource Profile: National Cancer Registration Dataset in England. Int J Epidemiol. 2020 Feb 1;49(1):16–16h.

13. ONS Open Geography Portal. Cancer Alliances (April 2020) Full Clipped Boundaries EN [Internet]. [cited 2023 Oct 2]. Available from: https://geoportal.statistics.gov.uk/
