# Supplementary material for: The comprehensive English National Lynch Syndrome Registry: development and description of a new genomics data resource
Source: eClinicalMedicine. 2024 Feb 7;69:102465. doi: 10.1016/j.eclinm.2024.102465 (PMC10864212; doi:10.1016/j.eclinm.2024.102465)
Supplement: GMSA Lynch Consortium Author List [file mmc2.docx]

# The comprehensive English National Lynch Syndrome Registry: development and description of a new genomics data resource

**GMSA Lynch Consortium Author List**

| **First Name** | **Surname** | **Regional Genetic Service** |
| --- | --- | --- |
| Jacqueline | Cook | Sheffield Clinical Genetics Service |
| Ruth | Armstrong | East Anglia Regional Genetics Service |
| Munaza | Ahmed | North East Thames Regional Genetics Service |
| Terri | McVeigh | North East Thames Regional Genetics Service |
| Bianca | DeSouza | North West Thames Regional Genetics Service |
| Anjana | Kulkarni | South East Thames Regional Genetics Service |
| Heirdre | Bezuidenhout | South West Thames Regional Genetics Service |
| Richard | Martin | Northern Region Genetics Service |
| Debbie | Holliday | Yorkshire Regional Genetics Service |
| Rachel | Hart | Cheshire and Merseyside Clinical Genetics Centre |
| Fiona | Lalloo | Manchester Regional Genetics Centre |
| Alan | Donaldson | Bristol Clinical Genetics Service |
| Ruth | Cleaver | Peninsula Clinical Genetics Service |
| Catherine | Willis | Wessex Clinical Genetics Service |
| Victoria | Kiesel | Leicester Clinical Genetics Service |
| Marie-Anne | O’Reilly | Nottingham Department of Clinical Genetics |
| Dorothy | Halliday | Oxford Regional Genetics Service |
| Joyce | Solomons | Oxford Regional Genetics Service |
| Kai Ren | Ong | West Midlands Clinical Genetics Service |
